# Schwann cells-derived exosomes promote functional recovery after spinal cord injury by promoting angiogenesis

**DOI:** 10.3389/fncel.2022.1077071

**Published:** 2023-01-04

**Authors:** Jiang-Hu Huang, Yong-Neng Chen, Hang He, Chun-Hui Fu, Zhao-Yi Xu, Fei-Yue Lin

**Affiliations:** ^1^Department of Orthopedics, Fujian Provincial Hospital, Fujian Medical University, Fuzhou, China; ^2^Fuzhou Maixin Biotech. Co., Ltd., Fuzhou, China

**Keywords:** Schwann cells, exosomes, angiogenesis, integrin-β1, spinal cord injury

## Abstract

Exosomes are small vesicles that contain diverse miRNA, mRNA, and proteins that are secreted by multiple cells, and play a vital function in cell–cell communication. Numerous exosomes produced by cells have been demonstrated to be protective against spinal cord injury (SCI). This study aims to investigate the neuroprotective effect of Schwann cells-derived exosomes (SCs-Exos) on spinal cord injury. We found that SCs-Exos can be taken directly by brain-derived endothelial cells.3 (bEnd.3 cells) and promoted to proliferate, migrate, and form bEnd.3 tube. Additionally, our results showed that the pro-angiogenesis molecules, Integrin-β1, were highly expressed in SCs-Exos. Moreover, we used special shRNA technology to investigate the role of Integrin-β1 in mediating the effect of SCs-Exos-induced angiogenesis on bEnd.3 cells. We observed that the pro-angiogenic effect of SCs-Exos on bEnd.3 cells was suppressed by inhibiting the expression of integrin-β1 in SCs-Exos. In the SCI model, we found that SCs-Exos attenuated tissue damage and improved functional recovery after SCI. Using immunofluorescence staining, we observed that SCs-Exos treatment promoted angiogenesis in SCI, and integrin-β1 was required to promote angiogenesis. In conclusion, our results indicate that SCs-Exos promote angiogenesis by delivering integrin-β1 and may serve as a promising novel therapeutic agent for enhancing neurological functional recovery after SCI.

## Introduction

Acute spinal cord injury (SCI), a condition frequently brought on by trauma, typically results in loss of movement and sensation, and places a huge burden on each patient’s family and social society healthcare system (Carlson and Gorden, 2002). Numerous medications have been shown to be useful in treating SCI in animal models due to advances in science and technology, but none of them have been successful in human clinical trials (Thuret et al., 2006). Therefore, it is critical to unravel the molecular mechanisms of SCI and develop new effective treatments for this devastating condition.

In the last decade, cell-based therapy has proven to be an effective treatment for SCI (Assinck et al., 2017). Schwann cells (SCs) are located in the peripheral nervous system. After SCI, SCs in the nerve roots could migrate to the injured site of the spinal cord (Takami et al., 2002). Spinal cord damage can be successfully treated by SCs transplantation, according to several studies (Takami et al., 2002; Oudega and Xu, 2006; Deng et al., 2015). Through the promotion of myelination and axonal regeneration, the transplantation of SCs exerts a protective impact following SCI (Deng et al., 2015). Immune rejection, however, limits the use of cell transplantation therapy (Assinck et al., 2017). According to a prior study, the therapeutic efficacy of SCs is mostly dependent on the neurotrophic factors and extracellular chemicals they produce (Oudega and Xu, 2006).

Recently, exosomes are a particular kind of extracellular vesicle that has attracted researchers’ attention. Exosomes are small vesicles (30–150 nm) containing various types of miRNA, mRNA, and protein. It has been demonstrated to be present in a wide range of tissues and cells and is crucial for cell–cell communication (Dreyer and Baur, 2016). There are several benefits to using exosomes in the therapy of illnesses. First, the exosomes have a stable, two-layer membrane structure that resists degradation. Second, relative to cell transplantation, exosomes are safer with no immunological rejection. Due to their tiny size, exosomes do not readily obstruct microvessels as other cells do. Exosomes, third, are nanoscale vehicles that can cross the plasma membrane. Studies have shown that exosomes can cross the plasma membrane as well as the blood–brain barriers with less immunological rejection (Record et al., 2011; Dreyer and Baur, 2016). Therefore, exosome-based therapy for central nervous system (CNS) diseases has attracted great attention from researchers (Goetzl et al., 2015; Lee et al., 2016; Zhang et al., 2017; Zheng et al., 2019). Exosomes from SCs have significantly improved axonal regeneration in the sciatic nerve damage model by reducing the activity of the GTPase RhoA (Lopez-Verrilli et al., 2013). A study by Pan et al. (2022) found that Schwann cells-derived exosomes (SCs-Exos) have a positive effect on promoting functional recovery after spinal cord injury in rats. In our present study, we aimed to investigate whether SCs-Exos has a neuroprotective effect after SCI and elucidate the underlying mechanism.

## Materials and methods

### Animals

Adult male Sprague–Dawley (SD) rats (180–220 g) were provided by the Animal Center of Fujian Provincial Hospital (Fuzhou, China). All animal care and experimental procedures were approved by the Ethics Committee of Fujian Provincial Hospital (NO 2018-02-013). All animals were housed in individual cages in a temperature- and light-cycle-controlled environment and given free access to food and water.

### Culture of rat SCs and identification

Following anesthesia with sodium pentobarbital (Sigma, St. Louis, MO, USA), the rats were sacrificed and immersed in 75% alcohol for 10 s for disinfection.

The sciatic nerve was removed under sterile conditions and washed with phosphate-buffered saline (PBS) two times, and cut into small pieces with ophthalmic scissors. The tissues were digested with 0.125% trypsin and 0.125% collagenase for 25 min followed by centrifuging at 1,500 rpm for 7 min. The supernatant was discarded, and 5 ml of PBS was added to suspend and wash the cells two times. Cells were collected and incubated in an incubator with 5% CO_2_ at 37° for 24 h. The next day cells were treated with IgM class anti-Thy 1.1 antibodies (Serotec, Kidling, UK) and rabbit complement (Sigma-Aldrich, St. Louis, MO, USA) to eliminate fibroblasts. The culture medium was replaced with fresh medium supplemented with Dulbecco’s modified eagle medium (DMEM)-10%, 20 mg/ml bovine pituitary extract (Invitrogen, Waltham, CA, USA), penicillin-streptomycin supplemented with 2 mM forskolin (Millipore, Burlington, MA, USA), and FBS-1%. After 7 days, SCs at the primary passage were detached with 0.25% EDTA–trypsin and passage. A routine cell culture method was then carried out, and the cells were identified by anti-S100 (rabbit 1:500, DakoCytomation, Santa Clara, CA, USA) and anti-P75 NTR (rabbit 1:500, Abcam, UK) immunostaining before the subsequent experiments according to previous description (Zheng et al., 2010).

### Culture of bEnd.3 cells

The bEnd.3 cells (brain-derived endothelial cells.3) were obtained from the Type Culture Collection of the Chinese Academy of Sciences (Shanghai, China). Cells were cultured in Dulbecco’s Modified Eagle’s Medium (DMEM) (Sigma-Aldrich, St. Louis, MO, USA), containing 4,500 mg glucose/L, 10% FBS, and 1% penicillin-streptomycin. The medium was changed every 3 days. Cells were maintained in a cell culture incubator at 37°C, 5% CO2, and a humidified atmosphere.

### The extraction and identification of exosomes

Schwann cells-derived exosomes were collected as previously described (Huang et al., 2017). After SCs were spread to the bottom of the cell culture flask for about 80%, were washed with PBS three times and the SCs serum-free medium was replaced, and the cells returned to the culture hood for 48 h. The cells supernatant was collected, centrifuged at 1,000 × *g* for 10 min at 4°C to remove residual cells, and centrifuged at 4°C 1,000 × *g* for 20 min to remove cell debris. The resulting supernatant was filtered using a 0.22 μm filter (Millipore, Burlington, MA, USA). Then, the filtered supernatant was centrifuged at 100,000 × *g* for 70 min at 4°C to obtain the exosomes. The supernatant was removed, 500 μL of PBS was used to dissolve the SC-Exos, and transferred to a sterile EP tube for subsequent experiments. The total protein concentration of exosomes was quantified using a Bradford assay (BioRad, Hercules, CA, USA). Transmission electron microscopy (TEM), nanoparticle tracking analysis, and Western blotting were used to identify exosomes.

## Oxygen glucose deprivation

After incubation with PBS, SCs^ctrl shRNA^-Exos, or SCs^shIntegrin–β1^-Exos, respectively, for 6 h, the bEnd.3 cells were then subjected to OGD. Briefly, the culture medium was replaced by glucose-free DMEM (Gibco, Hercules, CA, USA) and then placed into an anaerobic and humidified incubator, which was suffused with 94% N2, 1% O2, and 5% CO2 for 4 h at 37°C. Then, cells were washed two times with Roswell Park Memorial Institute (RPMI) 1,640 for two times and returned to a normal culture medium for 6 h to conduct further experiments.

### Exosomes uptake by bEnd.3 cells

To determine exosome uptake by bEnd.3 cells, a green fluorescent dye (PKH67, Sigma-Aldrich, St. Louis, Mo, USA) was used to label exosomes, according to the previous study (Huang et al., 2017). The resuspended exosomes were then stained with PKH67 using a labeling kit (Sigma-Aldrich, St. Louis, Mo, USA). PKH67 dye was diluted in 100 μL diluent C to a final concentration of 8 μM. Then, 10 μg of exosomes in 20 μL DPBS were diluted with 80 μL diluent C, added to the dye solution, and incubated for 5 min while mixed with gentle pipetting. Then, the labeled exosomes were diluted to 1 ml with PBS and pelleted by ultracentrifugation at 100,000 × *g* for 1 h 10 min at 4°. Then, labeled exosome co-cultured with bEnd.3 cells for 3 h at 37°C. Next, the cells were fixed in 4% PFA for 15 min at RT. The cells were then washed with PBS three times, followed by stained nuclei with DAPI (1:500, Invitrogen, Waltham, CA, USA) for 5 min at RT. The signal was finally observed by a fluorescence microscope.

### EdU test

The EdU test was used to determine how SCs-Exos affected the proliferation of bEnd.3 cells. Briefly, 1 × 105 cells were seeded by DMEM with 10% FBS supplemented for 24 h, then treated for 2 h with 10 m EdU-labeling reagent (1:1,000, Invitrogen, Waltham, CA, USA), followed by the addition of exosomes. The medium was removed and washed with PBS for 5 min two times. 30-min incubation in 4% PFA at room temperature (RT), 5-min incubation in glycine (2 mg/ml) at RT, and a 5-min wash in PBS. Cells were identified by following the manufacturer’s instructions for using the ClickiT Edu Alexa Fluor 555 Imaging Kit (Invitrogen, Waltham, CA, USA). Cell nuclei were stained with 4′,6-diamidino-2-phenylindole (DAPI, 1:500, Invitrogen, Waltham, CA, USA) for 5 min at room temperature. In total, 10 randomly selected fields were examined with a fluorescence microscope to determine the percentage of cells that were EdU-positive.

### The capillary network formation assay

After treated with PBS, SCs-Exos, SCs^ctrl shRNA^-Exos, or SCs^shIntegrin–β1^-Exos, respectively, for 4 h, and then OGD for 4 h, 1 × 10^5^/well bEnd.3 cells were seeded at a density of 3/well on top of Matrigel in a 96-well plate. The cells were allowed to develop networks in culture for 6 h. Furthermore, an inverted microscope was used to see the tube creation. Image-Pro Plus 6.0 was utilized to quantitatively analyze the network architecture (Media Cybernetics, Rockville, MD, USA).

### Transwell chamber migration assay

Brain-derived endothelial cells.3 cells pretreated with PBS, SCs-Exos, SCs^ctrl shRNA^-Exos, or SCs^shIntegrin–β1^-Exos, followed by OGD for 4 h. Cells of the bEnd.3 lines were cultured in serum-free media in the top chamber of a Matrigel-coated insert (BD, USA). The Transwell^®^ plate wells were filled with a migration-inducing material. A total of 1 × 10^5^ cells per well were placed in the top of the chambers. A total of 24 h later, cells remaining in the upper chambers were cleaned. Furthermore, 0.1% crystal violet was used to label the migrating cells, and an Olympus inverted microscope was utilized to tally the cell count.

### siRNA

To investigate the effect of integrin-β1 in SCs-Exos-induced angiogenesis on bEnd.3 cells, the integrin-β1-specific shRNA (sh Integrin-β1, n387129) and Control siRNA were purchased from Thermo Fisher Scientific to repress the expression of integrin-β1 in bEnd.3 cells. Briefly, SCs were transfected with Integrin-β1 shRNA or control shRNA (ctrl shRNA) using Lipofectamine 2,000 (Thermo Fisher Scientific, Waltham, MA, USA) according to the manufacturer’s instructions. The inhibition efficiency of this shRNA was measured by Western blotting and qRT-PCR after 48 h.

### qRT-PCR

Total RNA was extracted from bEnd.3 cells with TRIzol (Invitrogen, Waltham, CA, USA). Then, the RNA was reverse-transcripted into cDNA using the PrimeScript RT reagent Kit (TaKaRa, Tokyo, Japan). Furthermore, qRT-PCR was performed with the SYBR Premix Ex Taq (TaKaRa, Tokyo, Japan) on an ABI PRISM^®^ 7900HT System (Applied Biosystems, USA). Analyses of gene expression were performed by the delta–delta CT method. The GAPDH was used as an internal reference. The primers used in this study are as follows: Integrin-β1, F: 5′GTAACCAACCGTAGCAAA GGAACAGC-3′, R: 5′-ATGTCTGTGGCTCCCCTGATCTTA-3′; GAPDH: F: 5′-TGGGCTACACTGAGCACCAG-3′, and R: 5′-AAGTGGTCGTTGAGGGCAAT-3′.

## Western blotting

The total proteins were obtained from cells or spinal cord issues. A BCA protein assay kit (Beyotime, Shanghai, China) was used to measure the protein concentrations. Furthermore, the proteins were separated by a 10% SDS/PAGE and transferred to the PVDF membranes (Millipore, Burlington, MA, USA). Next, the membranes were blocked in 5% non-fat milk at RT for 1 h. The membranes were then incubated with primary anti-Integrin-β1 (1:1,000, Abcam), anti-Integrin-α1 (1:1,000, Abcam), and anti-VEGF-A antibody (1:200, Abcam) at 4°C overnight. It was washed with PBS and incubated with anti-mouse IgG horseradish peroxidase-conjugated secondary antibody (1:2,000; Jackson) at RT for 1 h. Then, the bands were visualized using an ECL kit (Beyotime, China). The GAPDH served as an internal control.

### Establishment of contusion SCI model in rats

Adult Sprague–Dawley rats (male, 180–220 g) were provided by the Animal Center of Fujian Provincial Hospital (Fujian, China). The animal experiments were performed in accordance with the Ethics Committee of Fujian Provincial Hospital. All procedures involving animals in this study were approved by the Ethics Committee of Fujian Provincial Hospital. All rats were housed in individual cages, in temperature and light-cycle-controlled environments, and they had unrestricted access to both food and water.

The rat contusion SCI model was established according to our previous study (Huang et al., 2017). Rats were anesthetized with sodium pentobarbital (Sigma, St. Louis, MO, USA), and routine skin preparation, disinfection, and paving. After the T10 spinous process as the center, the skin was cut in the median line, and the muscles were removed to reveal the T9-T11 spinous processes and lamina. The T10 spinous process and lamina were bitten and a circular area of approximately 3 mm in diameter centered on the spinal cord segment of T10 was revealed. Centered on the median blood vessel behind the spinal cord, 8 g striking rods were used to fall from the T10 spinal cord from a height of 4 cm. The injury to the spinal cord caused by the intramedullary hemorrhage was visible to the naked eye; in addition, the rat tail wiggled, and the lower limbs and the body retracted and fluttered. The wound was cleansed and the incision was closed in layers. The incision was exposed to complicated iodine disinfection (1 time per day for 3 days). Each rat’s quadriceps were alternately injected with 4WU/time/day of penicillin for 3 days. In the Sham operation group, just a laminectomy was performed.

## Experiment design

A total of 40 rats were randomly divided into four groups (*n* = 10 per group). In the Sham group, the rats were subjected to laminectomy only. In the Control group, the rats received SCI and then at 30 min after SCI, were treated with 0.5 ml of PBS by tail vein injection. In the SCI + SCs^ctrlshRNA^-Exos group and the SCI + SCs^shIntegrin–β1^-Exos group, the rats received SCI, and then at 30 min after SCI, were treated with 100 μg of exosomes precipitate in 0.5 ml of PBS by tail vein injection. At scheduled experiment time points, the rats were sacrificed after deep anesthesia. For subsequent experiments, 10 mm of spinal cord tissue, including the lesion center, was harvested.

## Immunohistochemistry

On day 7 after SCI, we analyzed the presence of blood vessels in the spinal cord with an immunohistochemistry kit according to the manufacturer’s protocol (Huang et al., 2020). After the spinal cord was harvested, the specimens were fixed with PFA for 24 h, and then dehydrated and embedded. Ten consecutive 10-μm transverse sections per millimeter along the spinal cord axis were cut and mounted onto charged microscope slides for primary rabbit anti-rat polyclonal CD31 antibody at 4°C overnight. Then, an immunohistochemistry kit was used for the staining. The nuclei were counterstained with hemalum. The positive-stained CD31 was quantified using Image-Pro Plus software 6.0 (Media Cybernetics, Rockville, MD, USA).

### Immunofluorescence staining

To investigate the angiogenesis role of SCs-Exos on the spinal cord after SCI in rats, immunofluorescence staining was used for detecting the number of proliferating blood vessels in the spinal cord. On day 7 post-injury, the spinal cord was obtained. Then, the spinal cord was fixed with 4% paraformaldehyde at room temperature (RT) for 3 h. Moreover, it was incubated with 6% sucrose/PBS overnight. Then, the tissues were embedded in an optimal cutting temperature compound (OCT) (Sakura Finetek, USA). 5-μm-thick sections were then obtained in each group (*n* = 5/group). The frozen sections were air-dried for 15 min at room temperature, and the sections were blocked with 10% normal goat serum in PBS for 1 h at RT. Moreover, the sections were incubated with a mouse monoclonal CD31 antibody (1:100; Abcam) or a rabbit monoclonal proliferating cell nuclear antigen (PCNA) antibody (1:200; Abcam) overnight at 4°C. Washed with PBS, the sections were followed by incubation with the secondary antibodies Alexa Fluor 488 goat anti-rabbit IgG (H + L) (1:1,000, Jackson, USA) or Cy3 goat anti-mouse IgG (H + L) (1:1,000, Jackson, USA) for 30 min at RT. Washed with PBS 5 min × 3 times, the sections were incubated with DAPI working solution (CST, USA) at RT for 15 min. The numbers of CD31/PCNA co-staining positively in the spinal cord were counted in five randomly selected fields in the anterior horn of the gray matter in the spinal cord section per rat.

## Behavioral assessment

The locomotor functional recovery of the rats post-SCI was accessed by the Basso, Beattie and Bresnahan (BBB) scores (Basso et al., 1995). Behavioral assessment was performed at 1, 3, 5, 7, 14, 21, and 28 days after SCI. The movement of the rats was observed by two independent and well-trained observers, and the score was recorded according to the BBB scores. In total, 5 min of the movement of each rat was observed and repeated three times. Finally, the average score of the two observers was analyzed.

### Lesion identification by H&E staining

At 28 days post-SCI, after the behavioral assessment, the rats were anesthetized with sodium pentobarbital (Sigma, St. Louis, MO, USA) and transcardial perfusion with 4% PFA. A 10-mm-long spinal cord was obtained for HE staining, including the injury epicenter. The spinal cord tissues were embedded in paraffin. The longitudinal sections of the spinal cords were obtained with a thickness of 5 μm. Then, these sections were stained with H&E. The quantification of the cavity areas was accessed by Image-Pro Plus 6.0 (Media Cybernetics, Rockville, MD, USA).

## Statistical analysis

All data were analyzed using SPSS (17.0; SPSS, Inc., Chicago, IL) statistical software. The data were shown as means ± standard deviation (SD). The BBB scores were performed by repeated measures analysis of variance, followed by the Bonferroni *post-hoc* corrections. A one-way analysis of variance (ANOVA) was used to compare the means of multiple groups. Moreover, the means between the two groups were measured by an independent sample *t*-test. *P* < 0.05 was considered statistically significant.

## Results

### 9.1. Characterization of SCs and derived exosomes

**FIGURE 1:**
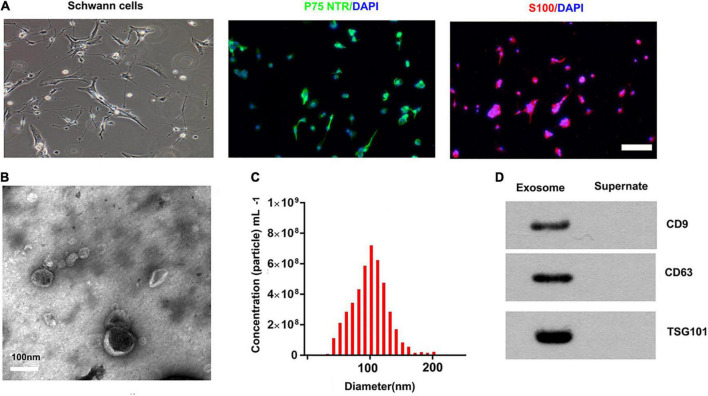
Characterization of SCs and derived exosomes. **(A)** Phase contrast image of the Schwann Cells (SCs). Immunofluorescence stain of the surfer markers-P75^NTR^ (green) and S100 (red) of SCs. Scale bar = 100 μm. **(B)** The Transmission electron micrograph of exosomes, Scale bar = 100 nm. **(C)** The nanoparticle tracking analysis of the exosomes. **(D)** The specific surface markers of exosomes, such as CD9, CD63, and TSG101 were then confirmed by Western blotting.

We used an immunofluorescence stain to confirm the surfer markers of SCs. Our results showed that the expression of P75^NTR^ and S100 was positive in these cells (Figure 1A). The TEM, nanoparticle tracking analysis, and Western blotting were used to access exosomes. Under the transmission electron microscope, the exosomes were a biconcave hemispherical structure, with a size of about 100 nm (Figure 1B). The nanoparticle tracking analysis showed that the size of exosomes ranged from 30 to 200 nm, peaking at 85 nm (Figure 1C). The specific surface markers of exosomes, such as CD9, CD63, and TSG101, were expressed in exosomes as confirmed by Western blotting (Figure 1D).

### 9.2. The SC-Exos have pro-angiogenic effects on endothelial cells

**FIGURE 2:**
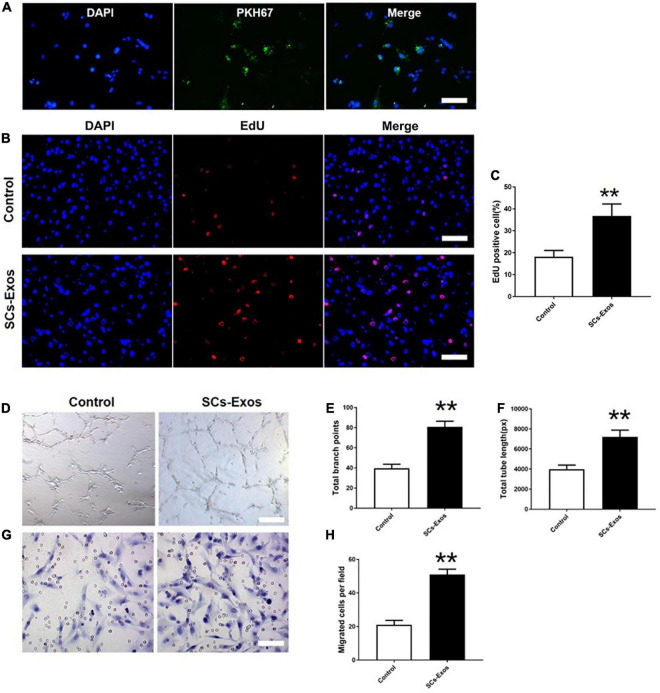
Schwann cells-derived exosomes enhance the angiogenic activities of Endothelial Cells. **(A)** Immunofluorescence images of PKH67-labeled exosomes taken up by bEnd.3 cells. Scale bar = 50 μm. **(B)** The proliferation of bEnd.3 cells was performed by the EdU test. **(C)** Quantitative analysis of EdU-positive cells in each group. **(D–F)** Tube formation and migration were measured by the capillary network formation assay and transwell chamber migration assay 6 h after seeding bEnd.3 cells pretreated with PBS, or SCs-Exos. Photomicrographs of tube-like structures and quantification of the tube number. **(G)** Photomicrographs of bEnd.3 cells that migrated through the Transwell^®^ membrane. The bEnd.3 cells were stained with crystal violet. **(H)** Quantification of the number of the migrated bEnd.3 cells. The data are presented as mean ± SD. **p < 0.01 SCs-Exos vs. Control group. *N* = 5 in each group. Scale bar = 500 μm.

To determine whether SCs-Exos could take up by bEnd.3 cells, we used PKH67 to label SCs-Exos. Then, added the labeled exosomes to the bEnd.3 cells. We observed that the PKH67 labeled exosomes were uptaken by endothelial cells and located in the cytoplasm of bEnd.3 cells (Figure 2A). These results revealed that bEnd.3 cells could take up SCs-Exos.

To assess the effect of SCs-Exos on the proliferation of bEnd.3 cells, the EdU test was performed. In the EdU test, there was a significantly higher percentage of EdU-positive cells in the SCs-Exos-treated groups than that in the Control group (*P* < 0.01; Figures 2B, C). The capillary network formation assay and transwell chamber migration assay were further conducted to access the pro-angiogenesis effects of SCs-Exos on bEnd.3 cells. In the capillary network formation assay, the number of total branch points and total tube length was higher in the SCs-Exos-treated groups than that in the Control group (*P* < 0.01; Figures 2D–F). Moreover, the migration of bEnd.3 cells in the SCs-Exos treated group was significantly greater than that in the Control group (*P* < 0.01; Figures 2G, H). These findings indicate that SC-Exos have pro-angiogenic effects on endothelial cells.

### 9.3. Identification of the pro-angiogenesis molecules in SCs-Exos

**FIGURE 3:**
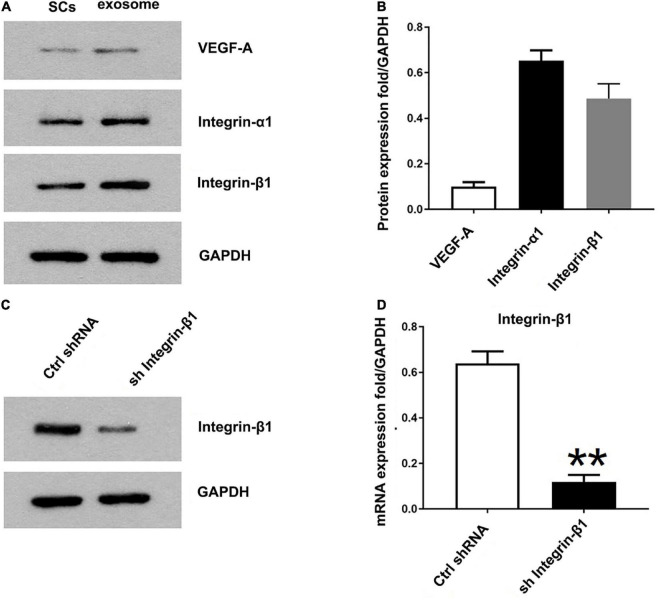
Identification of the pro-angiogenesis molecules in SCs-Exos and the inhibitory efficiency of integrin-β1 siRNA. **(A)** Western blotting was used to detect the protein level of VEGF-A, integrin-β1 and integrin-α1 in SCs and SCs-Exos. **(B)** The relative protein expression levels of VEGF-A, integrin-β1 and integrin-α1 in SCs-Exos **(C,D)**. Western blotting and qRT-PCR were performed to analyze the inhibitory efficiency of integrin-β1 siRNA. The data are presented as mean ± SD. **p < 0.01 sh Integrin-β1 group vs. ctrl shRNA group. *N* = 3 in each group.

Previously the study has identified 433 proteins cargo in SCs-Exos using proteomics analysis (Basso et al., 1995). We found that several proteins were closely related to angiogenesis, so we used a Western blot to confirm whether the angiogenesis molecular was highly expressed in SCs-Exos. Our results showed that two pro-angiogenesis molecules, integrin-β1, and integrin-α1 were highly expressed in SCs-Exos (Figures 3A, B). We also found that another pro-angiogenesis molecule, VEGF-A was expressed in SCs-Exos, but the expression level was relatively low (Figures 3A, B).

### 9.4. Integrin-β1 is required for SCs-Exos to promote angiogenesis on endothelial cells

**FIGURE 4:**
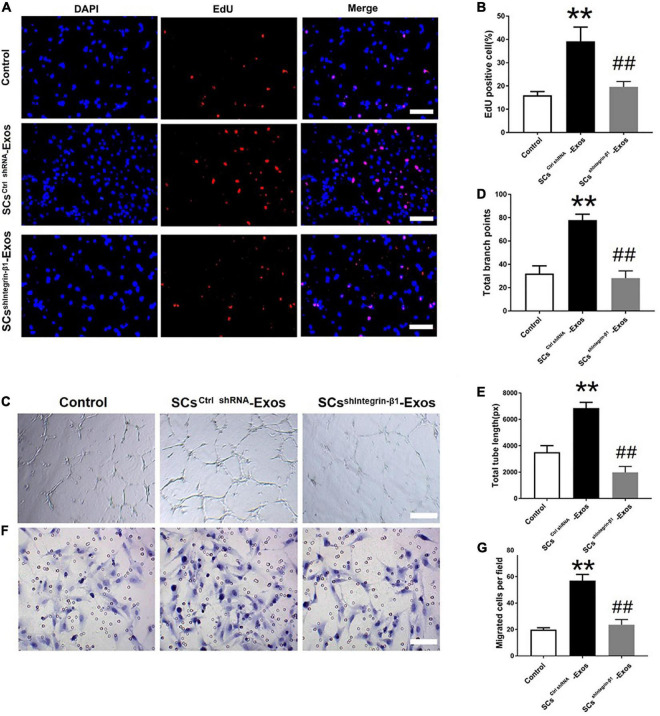
Integrin-β1 is required in mediating the role of SCs-Exos for promoting angiogenesis on Endothelial Cells. **(A)** The proliferation of bEnd.3 cells was performed by EdU test. **(D)** Quantitative analysis of EdU-positive cells in each group. **(B,E,F)** Tube formation and migration were measured by the capillary network formation assay and transwell chamber migration assay 6 h after seeding bEnd.3 cells pretreated with PBS, SCs^ctrlshRNA^-Exos, or SCs^sh^ ^Integrin–β1^-Exos. **(C)** Photomicrographs of tube-like structures and quantification of the tube number. Photomicrographs of bEnd.3 cells that migrated through the Transwell^®^ membrane. The bEnd.3 cells were stained with crystal violet. **(G)** Quantification of the number of the migrated bEnd.3 cells. The data are presented as mean ± SD. **p < 0.01 Control group vs. SCs^ctrlshRNA^-Exos group, ^##^*p* < 0.01 SCs^ctrlshRNA^-Exos group vs. SCs^shIntegrin–β1^-Exos group. *N* = 5 in each group. Scale bar = 500 μm.

To investigate the effect of Integrin-β1 on SCs-Exos induced angiogenesis on bEnd.3 cells, we repressed Integrin-β1 expression using an Integrin-β1-specific siRNA. Integrin-β1 silenced shRNA (sh Integrin-β1), and control shRNA (ctrl shRNA) were transfected into SCs, respectively. Then, exosomes were extracted from these transfected SCs. Our results showed that the Integrin-β1 protein and mRNA expression of SCs-Exos was greatly repressed by Integrin-β1 siRNA (*P* < 0.01; Figures 3C, D). In the EdU test, there was a significantly lower percentage of EdU-positive cells in the SCs^shIntegrin–β1^-Exos group than in the SCs^ctrlshRNA^-Exos group (*P* < 0.01; Figures 4A, D). The capillary network formation assay results showed that the number of total branch points and total tube length was significantly lower in the SCs^shIntegrin–β1^-Exos than in the SCs^ctrlshRNA^-Exos group (*P* < 0.01; Figures 4B, E, F). In the transwell chamber migration assay, the migration of bEnd.3 cells in the SCs^shIntegrin–β1^-Exos was significantly reduced than the SCs^ctrlshRNA^-Exos group (*P* < 0.01; Figures 4C, G).

### 9.5. Exosomal Integrin-β1 mediated the promoted angiogenesis effect of SCs-Exos in the injured spinal cord after SCI

**FIGURE 5:**
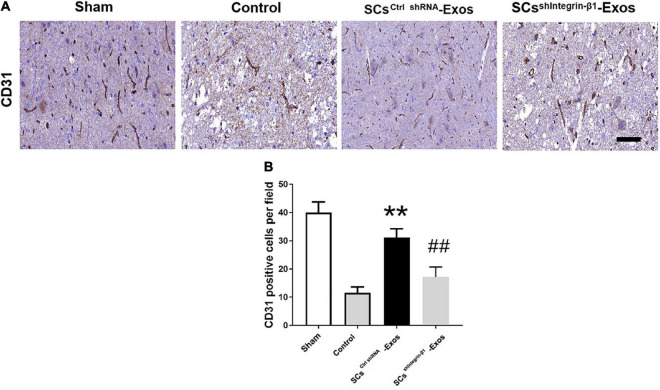
Exosomal Integrin-β1 of SCs-Exos promotes angiogenesis after SCI. **(A)** The presence of blood vessels in the spinal cord at day seven after SCI indifferent treated groups were analyzed by CD31. **(B)** Quantification of the number of CD31-positive cells in each group. The data are presented as mean ± SD. **p < 0.01 Control group vs. SCs^ctrlshRNA^-Exos group, ^##^*p* < 0.01 SCs^ctrlshRNA^-Exos group vs. SCs^sh^ ^Integrin–β1^-Exos group. *N* = 5 in each group. Scale Bar = 50 μm.

**FIGURE 6:**
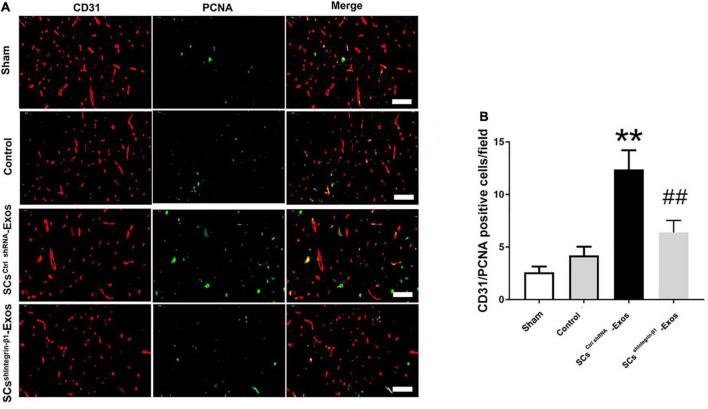
Exosomal Integrin-β1 of SCs-Exos promotes new blood vessel formation after SCI. The presence of proliferation of blood vessels in the spinal cord on day seven after SCI was analyzed by immunofluorescence staining. **(A)** Representative image of CD31-positive (Red) and PCNA-positive (Green) cells in the anterior horn of the spinal cord from each group at 7 days post-injury. **(B)** Quantitative analysis of CD31/PCNA-positive cells in the injured spinal cords from each group. The data are presented as mean ± SD. **p < 0.01 Control group vs. SCs^ctrlshRNA^-Exos group, ^##^*p* < 0.01 SCs^ctrlshRNA^-Exos group vs. SCs^shIntegrin–β1^-Exos group. *N* = 5 in each group. Bar = 50 μm.

CD31 staining was performed to evaluate the presence of blood vessels in the spinal cord at day 7 post-SCI. The results showed that the number of blood vessels in the Control group was significantly decreased when compared to the Sham group (*P* < 0.01; Figures 5A, B). Furthermore, SCs^ctrlshRNA^-Exos treatment significantly enhanced the number of blood vessels when compared with the Control group (*P* < 0.05; Figures 5A, B). But the pro-angiogenic effect was attenuated, once the Integrin-β1 was downregulated in SCs^shIntegrin–β1^-Exos treated group than that in SCs^ctrlshRNA^-Exos group (*P* < 0.05; Figures 5A, B). Immunofluorescence staining was employed to detect proliferating blood vessels in the spinal cord 7 days post-SCI after exosome therapy. The number of CD31/PCNA positive cells in the SCs^ctrlshRNA^-Exos treated group was greater than that in the Control group (*P* < 0.01; Figures 6A, B). Similarly, the number of CD31/PCNA positive cells in SCs^shIntegrin–β1^-Exos group was lower than that in SCs^ctrlshRNA^-Exos group (*P* < 0.01; Figures 6A, B). These data suggest that Integrin-β1 present in SCs-Exos is responsible for promoting angiogenesis after SCI.

### 9.6. Exosomal Integrin-β1 mediates the protective effects of SCs-Exos on neurological functional recovery after SCI

**FIGURE 7:**
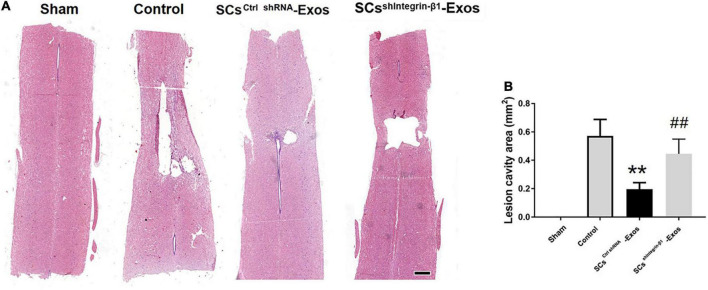
Exosomal Integrin-β1 of SCs-Exos attenuates tissue damage after SCI. **(A)** Representative H&E staining of lesion cavity of the spinal cord in each group. **(B)** Quantification of lesion cavity of each group. The data are presented as mean ± SD. **p < 0.01 Control group vs. SCs^ctrlshRNA^-Exos group, ^##^*p* < 0.01 SCs^ctrlshRNA^-Exos group vs. SCs^shIntegrin–β1^-Exos group. *N* = 5 in each group. Scale bar = 1 mm.

**FIGURE 8:**
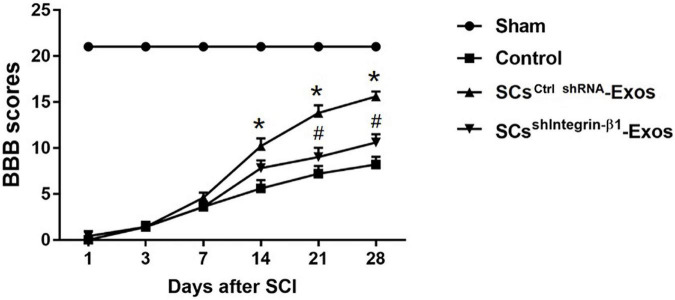
Exosomal Integrin-β1 of SCs-Exos improves neurological functional recovery after SCI. Functional recovery after SCI in rats was accessed by BBB scores, which ranged from Day 1 to Day 28 post-SCI. The data are presented as mean ± SD. *p < 0.05 Control group vs. SCs^ctrlshRNA^-Exos group, ^#^*p* < 0.05 SCs^ctrlshRNA^-Exos group vs. SCs^shIntegrin–β1^-Exos group. *N* = 5 in each group. Scale bar = 1 mm.

H&E staining was used to detect the histopathological changes in the injuries spinal cord with the treatment of exosome after SCI at day 28 post-injury. A huge lesion area was observed in the Control group, and SCs^ctrlshRNA^-Exos treatment significantly reduced the lesion area of the injured spinal cord (*p* < 0.01, Figures 7A, B). Moreover, the lesion area was larger in the SCs^shIntegrin–β1^-Exos treated group than that in SCs^ctrl shRNA^-Exos-treated group (*p* < 0.01, Figures 7A, B). The BBB scores were used to analyze the functional recovery after SCI. The results showed that the scores were 0 immediately after SCI in all groups, indicating that the SCI model in rats was produced successfully. Moreover, there was a spontaneous recovery of hind limb function in rats after SCI; the BBB score gradually increased in all treatment groups (Figure 8). At 14 days post-SCI, SCs^ctrlshRNA^-Exos treatment group had higher BBB scores than that in the Control group and SCs^shIntegrin–β1^-Exos group (*p* < 0.05, day 14, day 21, and day 28, Figure 8). Moreover, the BBB scores in the SCs^shIntegrin–β1^-Exos group were significantly lower than that in the SCs^ctrlshRNA^-Exos group since day 21 after SCI (*p* < 0.05, day 21, and day 28, Figure 8).

## Discussion

Spinal cord injury is a catastrophic disease. Effective treatment strategies for promoting functional recovery after SCI are still lacking. In the present study, we investigated the protective role of SCs-derived exosomes on functional recovery after SCI for the first time. Our results demonstrated that SCs-Exos treatment significantly promoted the proliferation, migration, and tube formation of endothelial cells. Moreover, we further confirmed that integrin-β1 was involved in these pro-angiogenesis processes and mediated the pro-angiogenesis effect of SCs-Exos on endothelial cells. As presented in the SCI model, SCs-derived exosomes enhanced microvascular density and pro-angiogenesis in the injured spinal cord after SCI. Furthermore, we observed that SCs-Exos treatment alleviated tissue damage of the spinal cord, and improved neurological functional recovery after SCI. Moreover, these protective effects of SCs-Exos on SCI were partly impaired by reducing the expression of integrin-β1 in exosomes cargo in SCs-Exos.

The pathophysiology of spinal cord injury is extremely complex and includes primary injury and secondary injury. Primary injury of the spinal cord was caused by immediate violence and cannot be avoided. Therefore, the focus of research is on how to reduce secondary injury of the spinal cord (Dumont et al., 2001). Studies have shown that the loss of vascular structure and the post-traumatic blood supply disturbance brought on by vascular dysfunction plays a significant role in exacerbating the spinal cord’s secondary damage and the loss of neurological function (Crock et al., 2005; Martirosyan et al., 2011). In this study, we found that the density of the microvascular in the spinal cord was decreased post-SCI, which was consistent with the previous report (Cao et al., 2017). However, the studies also demonstrated that the vessel could play a role in mediating nerve regeneration. A well vascular blood supply provides a sufficient nutrient substance for neural tissue survival. Therefore, how to reduce vascular damage and promote angiogenesis after SCI is crucial for promoting neurological functional recovery after SCI.

To date, multiple therapeutic interventions for promoting angiogenesis after SCI have been conducted. These treatments include proangiogenic factor administration, cell transplantation and gene therapy, and biomaterial implantation. The Schwann cells (SCs) are the glial cells of peripheral nerves, which can repair both peripheral nerve injury and CNS injury. The repair mechanism of SCs may be related to their secretion of cytokines and the extracellular matrix, which can improve the microenvironment of the injured site and promote angiogenesis and nerve regeneration (Takami et al., 2002; Webber and Zochodne, 2010; Lopez-Verrilli et al., 2013; Toft et al., 2013). Studies have shown that the transplantation of the Schwann cells can promote functional recovery after SCI, but the therapeutic effect was limited. The main reasons were due to its poor survival of SCs after transplantation and difficulty in crossing the blood–brain barrier in the injured spinal cord (Webber and Zochodne, 2010; Toft et al., 2013).

Exosomes have recently drawn a lot of attention from researchers for the treatment of CNS illnesses due to their tiny size and capacity to cross the blood–brain barrier (Braccioli et al., 2014; Zheng et al., 2019). Numerous studies have suggested that exosomes, which have a variety of roles including boosting angiogenesis and controlling inflammation, are essential for the healing of SCI. All cells have the capacity to produce exosomes, which serve the same function as their parent cells. In our current study, the pro-angiogenesis role of exosomes derived from the Schwann cells (SCs-Exo) was investigated in SCI and *in vitro* cell models. We co-cultured SCs-Exo with bEnd.3 cells and found that SCs-Exos could take up by bEnd.3 cells, and the SCs-Exos treatment group significantly promoted endothelial cell proliferation and migration and increased tube formation. These results suggest that SCs-Exos have pro-angiogenic effects on endothelial cells, which may be used as a therapeutic agent for enhancing angiogenesis after SCI.

Exosomes comprise a variety of substances, including miRNAs, mRNAs, and proteins. These contents of exosomal cargo could be delivered to their recipient cells through intercellular communication (Dreyer and Baur, 2016). Due to the lipid bilayer, exosomal contents could be protected against external proteases and other enzymes, and are relatively stable. A study analyzed and found that SCs-Exos contained 433 proteins, including a variety of proteins related to nerve regeneration, inflammation, and angiogenesis. Among the angiogenesis-related proteins, integrin-β1 and integrin-α1 were found to be enriched and expressed in SCs-Exos content (Wei et al., 2019). Integrin-β1 plays an important role in the injury of various tissues and organs and controls VE-cadherin localization and blood vessel stability (Milner and Campbell, 2002; Mettouchi and Meneguzzi, 2006; Keely et al., 2009; Malan et al., 2010; Goto et al., 2016). In this study, we further explored the role of integrin-β1 in mediating the pro-angiogenesis effects of SCs-Exos following SCI. It has been reported that the contents of exosomes could be modified by multiple genetic engineering technologies (Didiot et al., 2016; Zhao et al., 2019). We generated integrin-β1-down expression SCS-Exos from sh integrin-β1-transfected SCs. We found that compared with SCs^ctrlshRNA^-Exos, the level of integrin-β1 was downregulated in SCs^sh^ ^integrin–β1^-Exos. And the proliferation, migration, and tube formation of bEnd.3 cells were inhibited in the SCs^sh^ ^integrin–β1^-Exos group once the expression level of integrin-β1 was downregulated in the exosome derived from the genetically modified SCs. Our data also indicated that integrin-β1 can be transferred from SCs-Exos to bEnd.3 cells, and SCs-Exos exerted a pro-angiogenesis role on bEnd.3 cells partly by transferring integrin-β1.

We further investigated the role of SCs-Exos on the injured spinal cord after SCI and we injected SCs-Exos through the tail vein in the SCI model. We found that the microvascular density and the number of new blood vessel formations in the spinal cord tissue were significantly increased in the SCs-Exos-treated group, and the cavities of the spinal cord tissue in the SCs-Exo treatment group were significantly reduced compared with the Control groups, and the BBB score was significantly higher in SCs-Exo treatment groups than that in other injury groups. These demonstrated that SCs-Exo significantly improves angiogenesis, reduces tissue damage, and promotes the recovery of hindlimb function after SCI. These data also indicated that there is a close relationship between pro-angiogenesis and improved functional outcomes after SCI. However, these protective effects on the spinal cord were partly abolished when integrin-β1 was inhibited in SCs-Exo cargo. These results indicated that SCs-Exo exerted a pro-angiogenesis role on SCI partly due to its exosomal integrin-β1. However, this pro-angiogenesis effect of SCs-Exos was not completely blocked by integrin-β1 inhibition. This suggests that SCs-Exos also contain other molecules that play a role in promoting angiogenesis. Therefore, the role of other molecules in mediating the effect of SCs-Exos on promoting angiogenesis needs to be further investigated.

## Conclusion

In conclusion, in the present study, we revealed that SCs-Exos treatment promoted proliferation, migration, and tube formation of endothelial cells, promoted angiogenesis, reduced tissue damage, and facilitated neurological function recovery after SCI, and integrin-β1 played a crucial role in mediating the role of SCs-Exos in this process. Therefore, the upregulation of integrin-β1 in SCs-Exos may serve as a new potential therapeutic strategy for the treatment of SCI.

## Data availability statement

The original contributions presented in this study are included in the article/supplementary material, further inquiries can be directed to the corresponding author.

## Ethics statement

The animal study was reviewed and approved by the Ethics Committee of Fujian Provincial Hospital (license no. K2018-02-012).

## Author contributions

F-YL designed the study. J-HH, Y-NC, HH, and C-HF did the experiment and analyzed the data. J-HH and Z-YX analyzed the data and wrote the manuscript. All authors contributed to the article and approved the submitted version.
